# Magnetic Immobilization of *Pichia pastoris* Cells for the Production of Recombinant Human Serum Albumin

**DOI:** 10.3390/nano10010111

**Published:** 2020-01-06

**Authors:** Seyedeh-Masoumeh Taghizadeh, Alireza Ebrahiminezhad, Mohammad Bagher Ghoshoon, Ali Dehshahri, Aydin Berenjian, Younes Ghasemi

**Affiliations:** 1 Department of Pharmaceutical Biotechnology, School of Pharmacy and Pharmaceutical Sciences Research Center, Shiraz University of Medical Sciences, Shiraz, Iran; taghizm@sums.ac.ir (S.-M.T.); ghoshoon@gmail.com (M.B.G.); dehshahri@sums.ac.ir (A.D.); 2 Department of Medical Nanotechnology, School of Advanced Medical Sciences and Technologies, Shiraz University of Medical Sciences, Shiraz, Iran; a_ebrahimi@sums.ac.ir; 3 School of Engineering, Faculty of Science and Engineering, the University of Waikato, Hamilton 3240, New Zealand

**Keywords:** cell immobilization, cell harvesting, magnetite (Fe_3_O_4_) nanoparticles, magnetic separation, recombinant protein production

## Abstract

Magnetic immobilization as a novel technique was used to immobilize recombinant *Pichia pastoris* (GS115 Albumin) cells to produce human serum albumin (HSA). In this regard, magnetic nanoparticles (MNPs) coated with amino propyl triethoxy silane (APTES) were synthesized. *P. pastoris* cells were decorated with MNPs via nonspecific interactions. Decorated cells were magneto-responsible and easily harvested by applying an external magnetic field. The efficiency of magnetic immobilization (E_i_) for cell removal was in direct relation with the MNP concentration and time of exposure to the magnetic field. By increasing the nanoparticles concentration, cells were harvested in a shorter period. Complete cell removal (E_i_ ≈ 100) was achieved in ≥0.5 mg/mL of MNPs in just 30 s. HSA is produced in an extremely high cell density (OD ~20) and it is the first time that magnetic immobilization was successfully employed for harvesting such a thick cell suspension. After 5 days of induction the cells, which were immobilized with 0.25 to 1 mg/mL of nanoparticles, showed an increased potency for recombinant HSA production. The largest increase in HSA production (38.1%) was achieved in the cells that were immobilized with 0.5 mg/mL of nanoparticles. These results can be considered as a novel approach for further developments in the *P. pastoris*-based system.

## 1. Introduction

Methylotrophic yeast, *Pichia pastoris,* is one of the most important and interesting eukaryotic microorganisms in the biotechnological process. Recently, the production of recombinant proteins in *P. pastoris* has gained increasing attention in pharmaceutical and biotechnological industries. In contrast to other prokaryotic and eukaryotic systems, this expression system provides outstanding advantages, such as post-translation modifications, proper protein folding, ease of manipulation, high expression level, and low processing cost [1,2,3]. Lactoferrin, human growth hormone (hGH), insulin, human serum albumin (HSA), hepatitis B vaccine, interferon alpha 2b (hIFN-2b), heparin-binding EGF-like growth factor (HB-EGF), and phospholipase A are just examples of several recombinant biopharmaceuticals that are produced by *P. pastoris* [4,5,6,7,8].

In every biotechnological setting, cell harvesting is the high-cost and critical point in downstream processing. Centrifugation is the most common approach in this regard. In the pilot and industrial scales, this technique requires expensive equipment with high energy consumption and scheduled repair and maintenance services [9,10]. Hence, there is a vast attempt to develop cell immobilization techniques as an efficient alternative for centrifugation. The *P. pastoris* system is not exceptional and several attempts were done to efficiently immobilize this cell for the production of valuable products, such as HSA, alcohol oxidase, l-alanyl-l-glutamine, and invertase [11,12,13,14].

In common immobilization approaches, cells were entrapped in a polymer matrix or attached on to a carrier surface. Calcium alginate, polyacrylamide gels, hydroxyapatite ceramics, silicone polymers, and silica are the most common polymers that were commonly used for immobilization techniques [13,14,15,16,17]. In addition to facilitated cell manipulation, immobilization techniques can protect cells from harsh physical and chemical conditions, such as in dehydration-rehydration treatments and toxic materials [18,19]. Polymer networks make a barrier around the cells and cells encounter a microenvironment that is different from the production media. This microenvironment puts the immobilized cells in a lower metabolic activity with reduced production efficiency [20]. On the other hand, in some cases, the employed polymer can be toxic to the cells [13].

Magnetic immobilization is a very recent approach in cell immobilization that employs magnetic nanoparticles (MNPs) to make the cells magneto-responsible. Cells are simply decorated with MNPs via nonspecific interactions and can be harvested in an external magnetic field. In this setting, cells do not encounter a microenvironment, such as what occurs in polymer-based immobilization methods. Therefore, magnetic immobilization gathers the advantages of submerged fermentation and immobilization techniques [21,22,23]. In addition, decoration with MNPs increase the cells’ permeability and hence increase transport across the cytoplasmic membrane [24]. Recent investigations show that this phenomenon gives the cells high performance in contrast to intact cells [21,22,25]. Magnetic immobilization provides other advantages and can solve major problems in the biotechnological process. Biofilm formation can be reduced by the decoration of microbial cells with certain concentrations of MNPs [26]. Interestingly, it was shown that magnetic immobilization can also protect valuable industrial strains from viral infections [27].

Magnetic immobilization was employed to produce natural and recombinant products, such as menaquinone-7 and recombinant asparaginase (II), in bacterial cells [22,25]. In yeast cells, this technique was employed previously for magnetic modification of *Saccharomyces*, *Kluyveromyces*, *Rhodotorula*, and *Yarrowia* [28]. Also, some efforts have been done to separates *S*. *cerevisiae* waste biomass in the bottle neck of the final wine product [29]. Magnetic immobilized *S. cerevisiae* were used successfully for sucrose hydrolysis, hydrogen peroxide decomposition, and dye removal [30]. In this experiment, for the first time, magnetic immobilization was employed successfully to produce recombinant protein, HSA, in the *P. pastoris* system as a promising eukaryotic host. In this regard magnetite (Fe_3_O_4_) nanoparticles with (3-Aminopropyl)triethoxysilane (APTES) were employed to immobilize yeast cells. This coating was selected based on previous investigations that introduced APTES as a perfect coating to protect and stabilize magnetite nanoparticles [31]. In addition, this molecule provides amino groups on the nanoparticles that seems to improve the interactions with cells’ surface. However, it has been shown that nanoparticles with diverse functionalities can efficiently decorate microbial cells [32]. Therefore, it is critical to consider the results of the present study for further developments in recombinant *P. pastoris* technology.

## 2. Materials and Methods

### 2.1. Materials

Ferric chloride hexahydrate (FeCl_3_·6H_2_O), ferrous sulphate heptahydrate (FeSO_4_·7H_2_O), ammonia solution 32%, and absolute ethanol were purchased from Merck chemicals (Darmstadt, Hessen, Germany). (3-Aminopropyl) triethoxysilane (APTES) was obtained from Sigma-Aldrich (St. Louis, MO, USA). All chemicals were analytical grade and used as received without any further modifications. Millipore water (resistance >18 MΩ cm^−1^) was used throughout the experiment. Recombinant *P. pastoris* (GS115 Albumin) cells producing extracellular HSA were provided by Invitrogen (Invitrogen Corporation, San Diego, CA, USA).

### 2.2. Synthesis of Nanoparticles

MNPs were prepared by coprecipitation of ferric and ferrous ions in aqueous solution as described previously. In brief, 0.74 g of ferrous sulphate heptahydrate and 1.45 g of ferric chloride hexahydrate (molar ratio 1:2) were dissolved in 50 mL of deionized water under a nitrogen atmosphere at 70 °C and the solution was vigorously stirred for 1 h. Afterward, 5 mL of ammonia solution (32%) was rapidly injected to the mixture and stirring was continued for another 1 h. The resulting precipitate was washed three times with distilled water and dried in an oven at 50 °C.

APTES-coated MNPs were obtained by chemical modification of nanoparticles. In brief, 0.7 g of naked nanoparticles were dispersed in 25 mL of ethanol/water solution (volume ratio 1:1). APTES (2.8 mL) was added to the dispersion under nitrogen protection. The mixture was exposed to the ultrasonic bath for 10 min at 40 °C. The reaction was continued with stirring for another 2 h. The resulting prepared precipitate was washed with absolute ethanol and distilled water three times and dried in an oven at 50 °C.

### 2.3. Nanoparticles Characterization

The visual appearance of the prepared nanoparticles was evaluated by using transmission electron microscopy (TEM, Zeiss EM10C, HT 80 kV, Oberkochen, Germany). The particle size was measured by using ImageJ software version 1.47v, an image analysis software developed by the National institute of Health (NIH) (available at http://imagejnihgov/ij/), and analyzed with SPSS 25 (SPSS Inc., Chicago, IL, USA). Prepared nanoparticles were dispersed in deionized water (0.1 mg/mL) and zeta potential measurements were performed at room temperature by Malvern, Zetasizer Ver. 7.11 (Malvern, Worcestershire, UK). The nanoparticles’ crystal structure was evaluated by X-ray powder diffractometry (XRD, Siemens D5000, Karlsruhe, Germany). The chemical structure and functional groups on the iron nanoparticles were analyzed using Fourier transform infrared spectroscopy (FTIR, Perkin–Elmer, Überlingen, Germany) via KBr pellets. A vibrating sample magnetometer (VSM, Lake Shore 7404, Lake Shore Cryotronics, Inc., USA) was used to measure the magnetic properties of the particles at room temperature.

### 2.4. Cell Immobilization

Cell preparation was performed based on the Pichia expression kit manual (Protein expression, Catalog No. K1710-01, Version F, Invitrogen, San Diego, CA, USA) with some modifications. In this regard, *P. pastoris* cells were cultured in 50 mL of YPD (yeast peptone dextrose) broth to reach OD 4. The cells were washed and suspended in 10 mL of normal saline. MNPs, which were dispersed in an equal volume of normal saline, were added to the cell suspension. Immediate shaking was employed to disperse the nanoparticles over the cells. After 15 min, cells were harvested and suspended in 10 mL of fresh YPD media to obtain a cell density equal to OD ~20. This cell density is the recommended density for recombinant protein production in *P. pastoris*.

The efficiency of magnetic immobilization to harvest yeast cells was evaluated in various concentrations of MNPs. Immobilized cells were exposed to a magnetic field (1.4 T neodymium magnet) for various time periods and the immobilization efficiency was calculated via Equation (1):

(1) $$E_{i}=\frac{\mathrm{Cells}/{\mathrm{mL}}_{t0}-\mathrm{Cells}/{\mathrm{mL}}_{t1}}{\mathrm{Cells}/{\mathrm{mL}}_{t0}}\;\times \;100,$$

where E_i_ is the immobilization efficiency, and Cells/mL_t0_ and Cells/mL_t1_ are cells per mL before and after exposure to magnetic field, respectively.

### 2.5. Recombinant Protein Production

The potential of the free and immobilized *P. pastoris* cells for the production of recombinant HSA was evaluated. Induction of the cells was performed according to Pichia expression kit manual (Protein expression, Catalog No. K1710-01, Version F, Invitrogen, San Diego, CA, USA). In this regard, methanol (0.05% final concentration) was added daily to induct albumin production over 6 days. Production of recombinant HSA was monitored over 6 days of induction by using SDS-page densitometry.

### 2.6. Nanoparticles Interaction with the Cells

Interaction of MNPs with *P. pastoris* cells were visualized by scanning electron microscopy (SEM). A thin smear of the cells was spread on a glass slide and dried at room temperature. The specimens were fixed by passing them through the flame of a Bunsen burner and incubating them in 2.5% glutaraldehyde for 45 min. The slides were washed in normal saline for 15 min and dehydrated in serial concentrations of ethanol (30%, 50%, 70%, 80%, and 90%) for 10 min. The final dehydration step was performed in absolute ethanol for 20 min. To completely remove ethanol, slides were incubated at room temperature overnight.

## 3. Results

### 3.1. Nanoparticle Characterization

**Figure 1:**
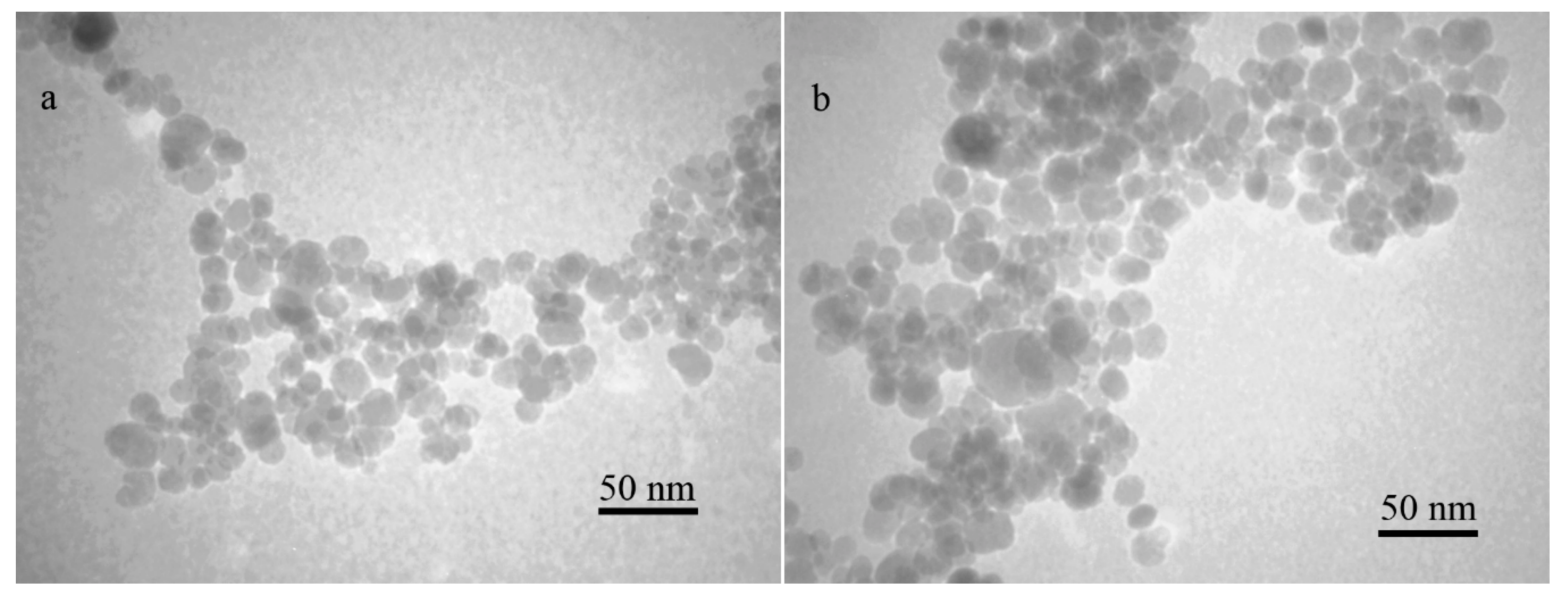
TEM micrographs of naked (**a**) and APTES-coated (**b**) magnetite nanoparticles.

**Figure 2:**
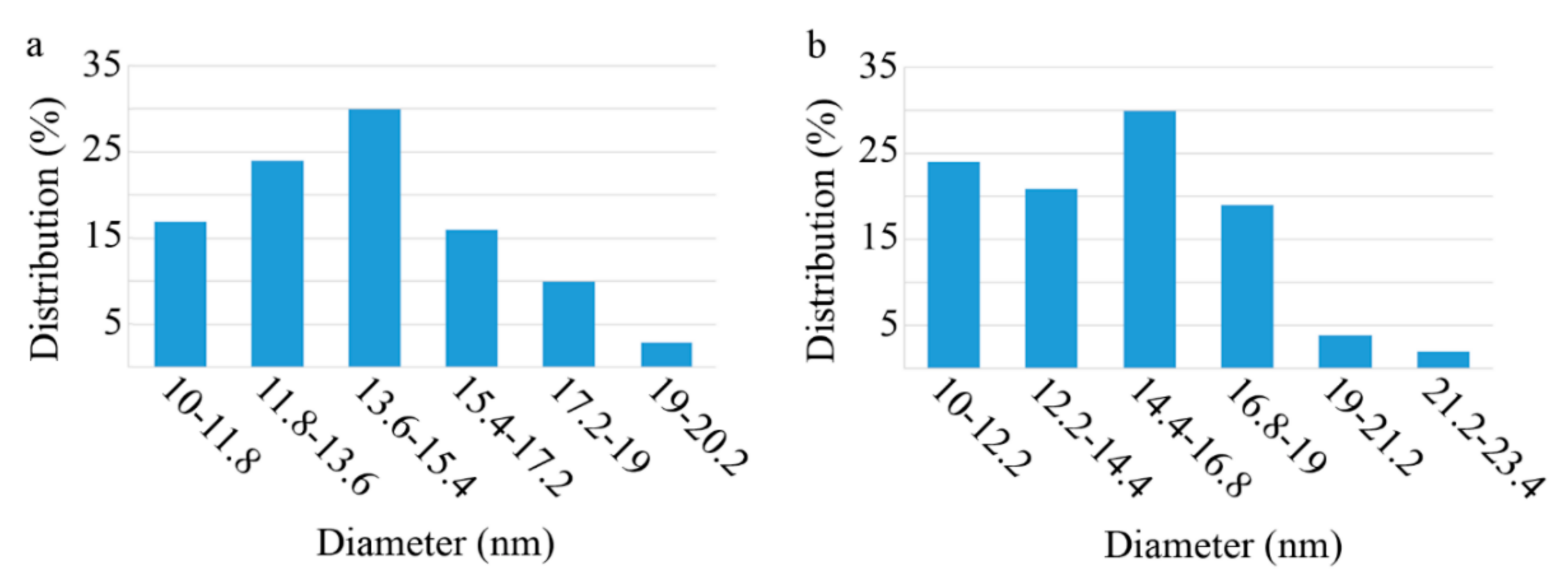
Particle size distribution histograms ((**a**): naked and (**b**): APTES coated) prepared from TEM micrographs by using ImageJ, an image processing program, and SPSS.

A TEM micrograph of the prepared nanoparticles is provided in Figure 1. The particles were found to be spherical in shape, with a narrow particle size distribution. The diameter of the naked particles ranged from 10 to 20 nm, with 14 nm being the mean particle size (Figure 2a). Meanwhile, APTES-coated particles showed a slight increase in size and measured to be 10 to 22.5 nm in diameter, with a 14.7 nm mean size (Figure 2b). This increase in size cannot be considered as significant and based on previous reports that APTES coating would not significantly increase the particles size distribution and mean particle size [33,34,35].

**Figure 3:**
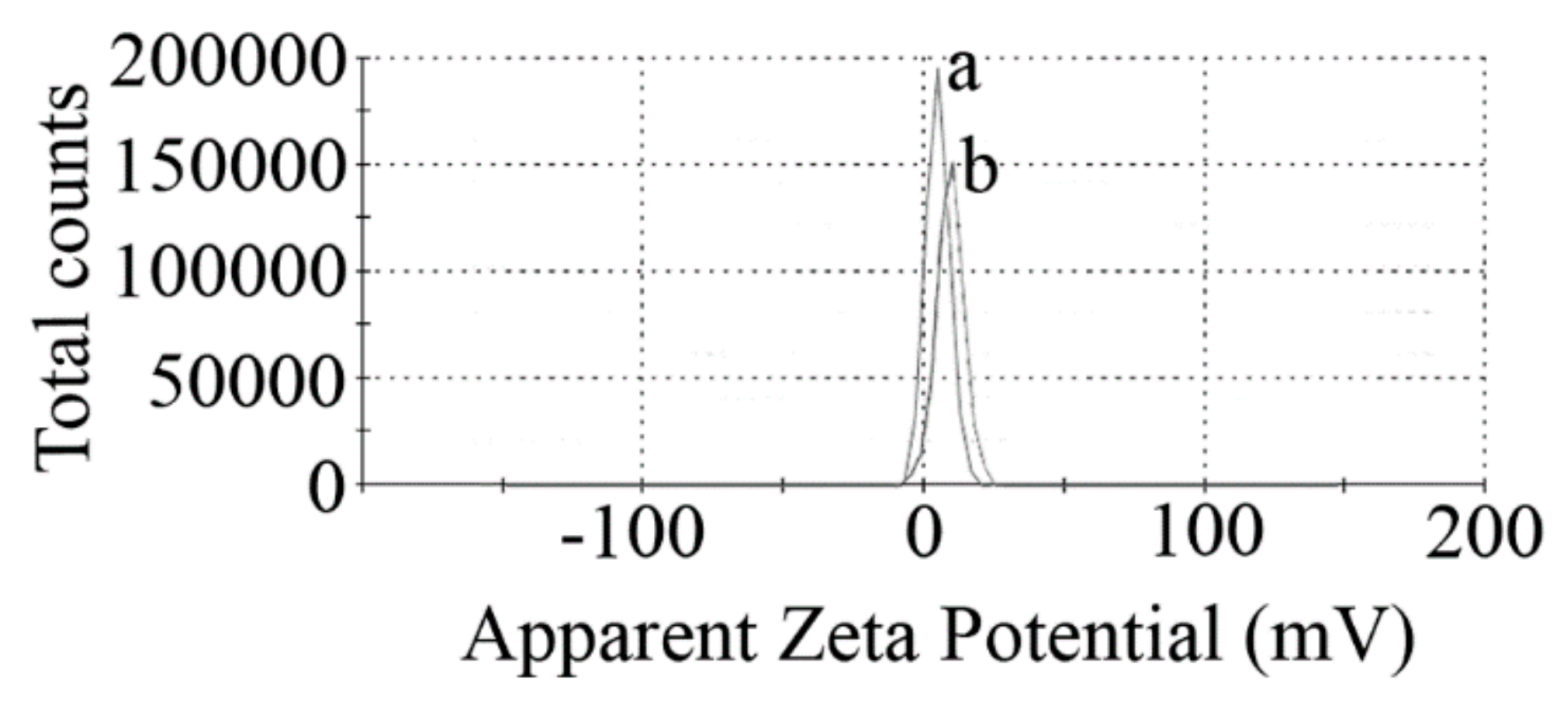
Surface charges (zeta potentials) of naked (**a**) and APTES-coated (**b**) MNPs.

The amino modification increases the zeta potential of MINPs from 4.77 to 8.28 mV (Figure 3).

**Figure 4:**
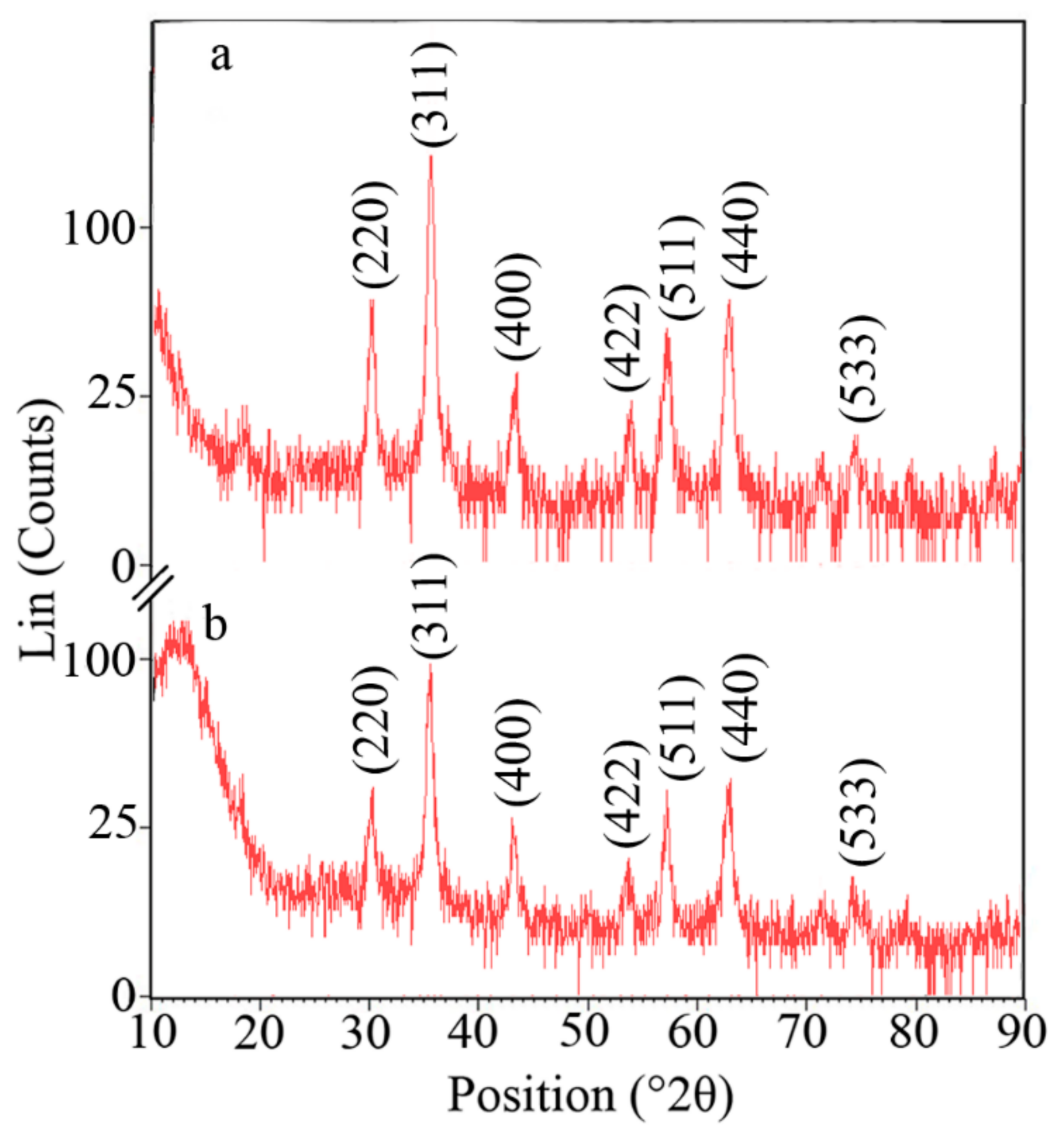
XRD patterns of naked (**a**) and APTES-coated (**b**) MNPs indicating the characteristic diffraction pattern of magnetite crystals.

The crystal structure of synthesized nanoparticles was analyzed using XRD in the range of 10 to 90 degrees of 2 theta angle. The XRD pattern of naked and APTES-coated nanoparticles showed characteristic peaks of magnetite crystals at 30.2°, 35.5°, 43.2°, 53.7°, 57.3°, 62.8°, and 74.3° of 2 theta values (Figure 4).

**Figure 5:**
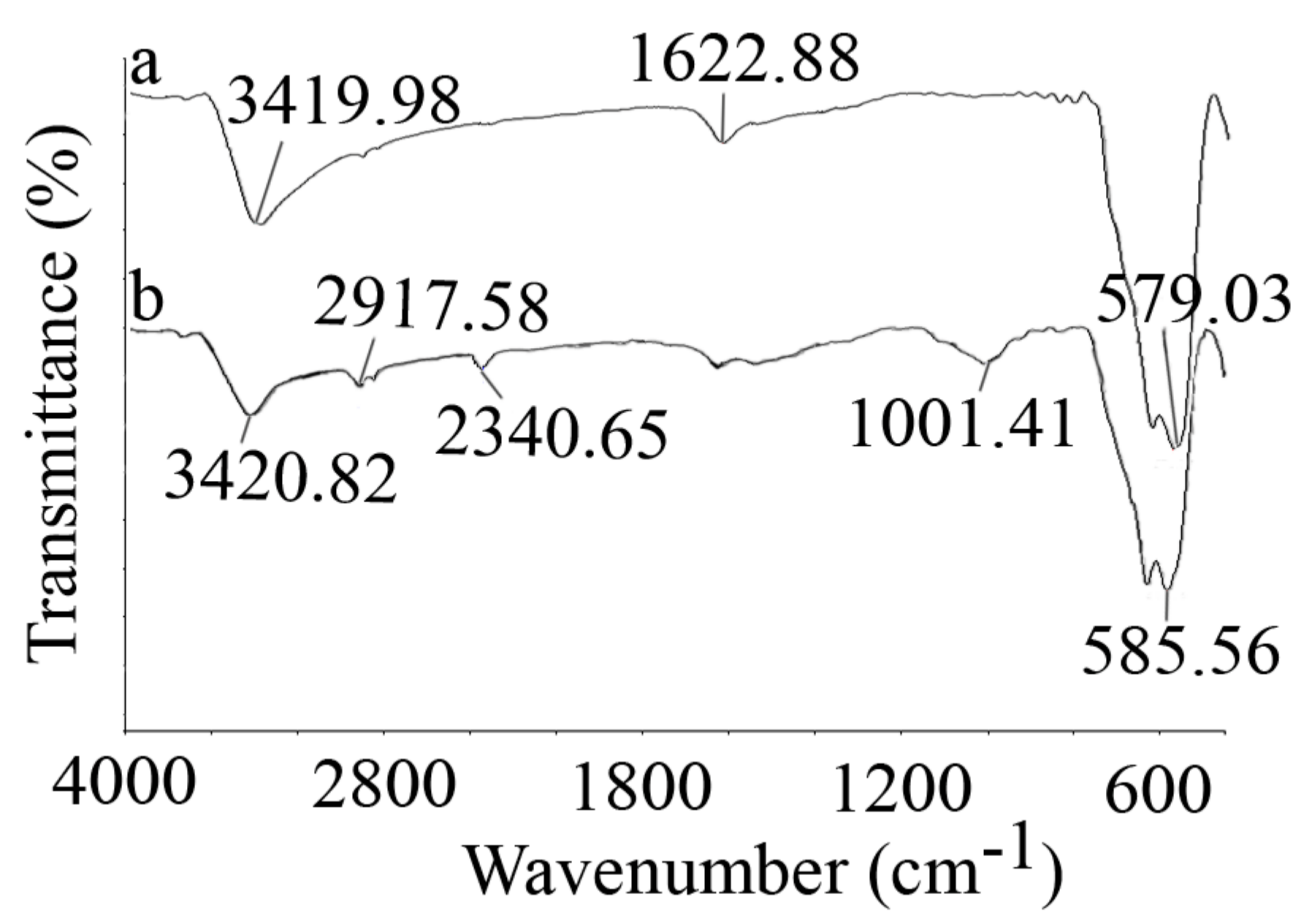
FTIR spectra of naked (**a**) and APTES-coated (**b**) MNPs, which were recorded via KBr pellets.

The FTIR spectra of the synthesized nanoparticles are illustrated in Figure 5. Strong Fe-O stretching vibration was observed at about 582 cm^−1^ for both naked and APTES-coated nanoparticles. The surface OH group depicted two bands at about 3420 (stretching) and ~1623 cm^−1^ (deforming) in both the naked and coated nanoparticles. The APTES coating on the surface of the particles was confirmed by the peaks at 1001.41 and 2340.65 cm^−1^, which are due to Si-O stretching vibration and Si-H bands, respectively.

**Figure 6:**
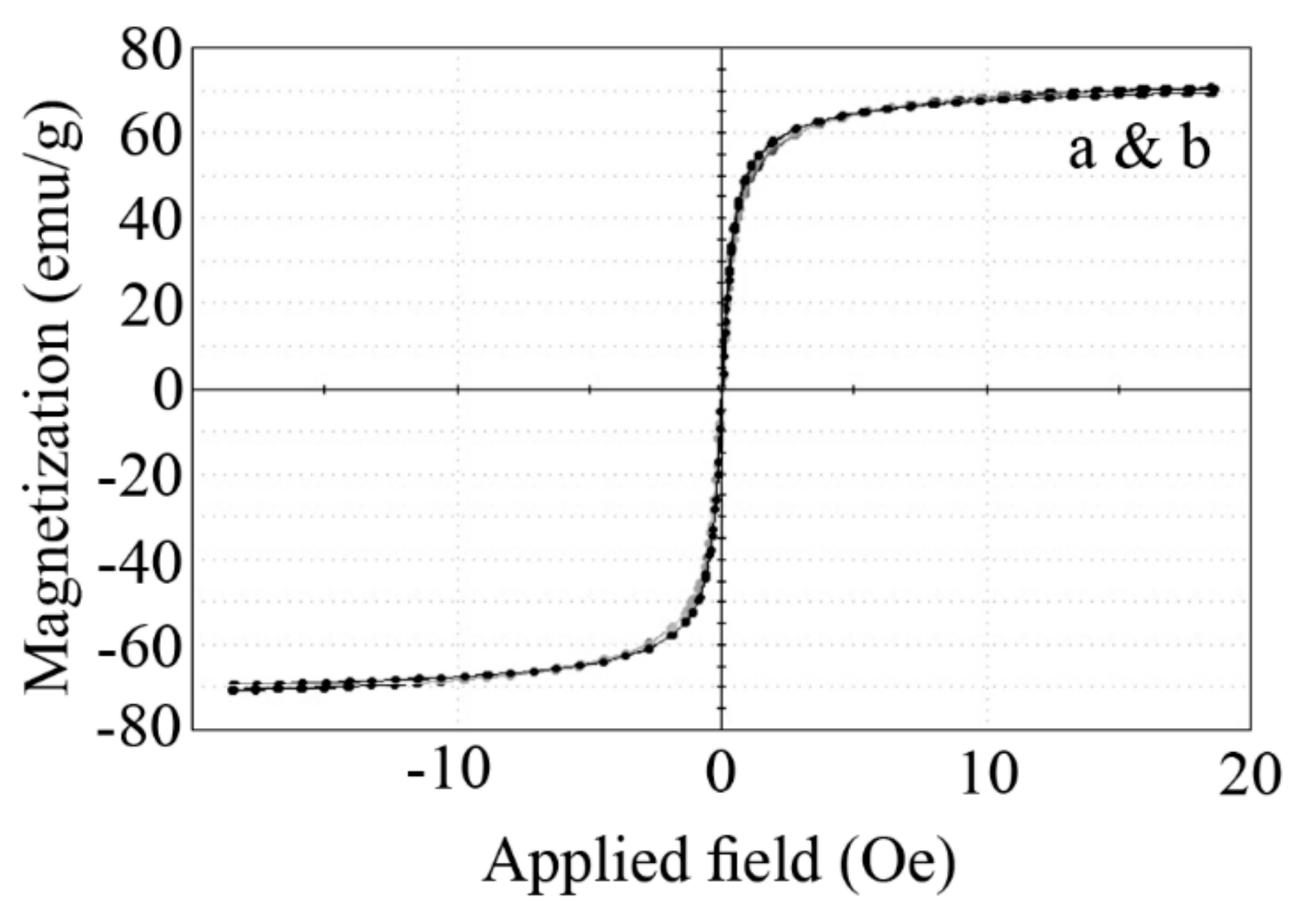
Magnetization curve (VSM) of naked (**a**) and APTES-coated (**b**) MNPs at room temperature.

The magnetization curves of naked and APTES-coated nanoparticles showed no hysteresis and were completely reversible (Figure 6). No coercivity and reduced remanence were observed, which indicates the superparamagnetic nature of the nanoparticles. The saturation magnetization was recorded to be 70.492 and 69.598 emu/g for naked and APTES-coated particles, respectively. These results were in close agreement with previous reports that the APTES coating has no significant effect on the magnetic properties of MNPs [36,37,38].

### 3.2. Cell Immobilization

**Scheme 1:**
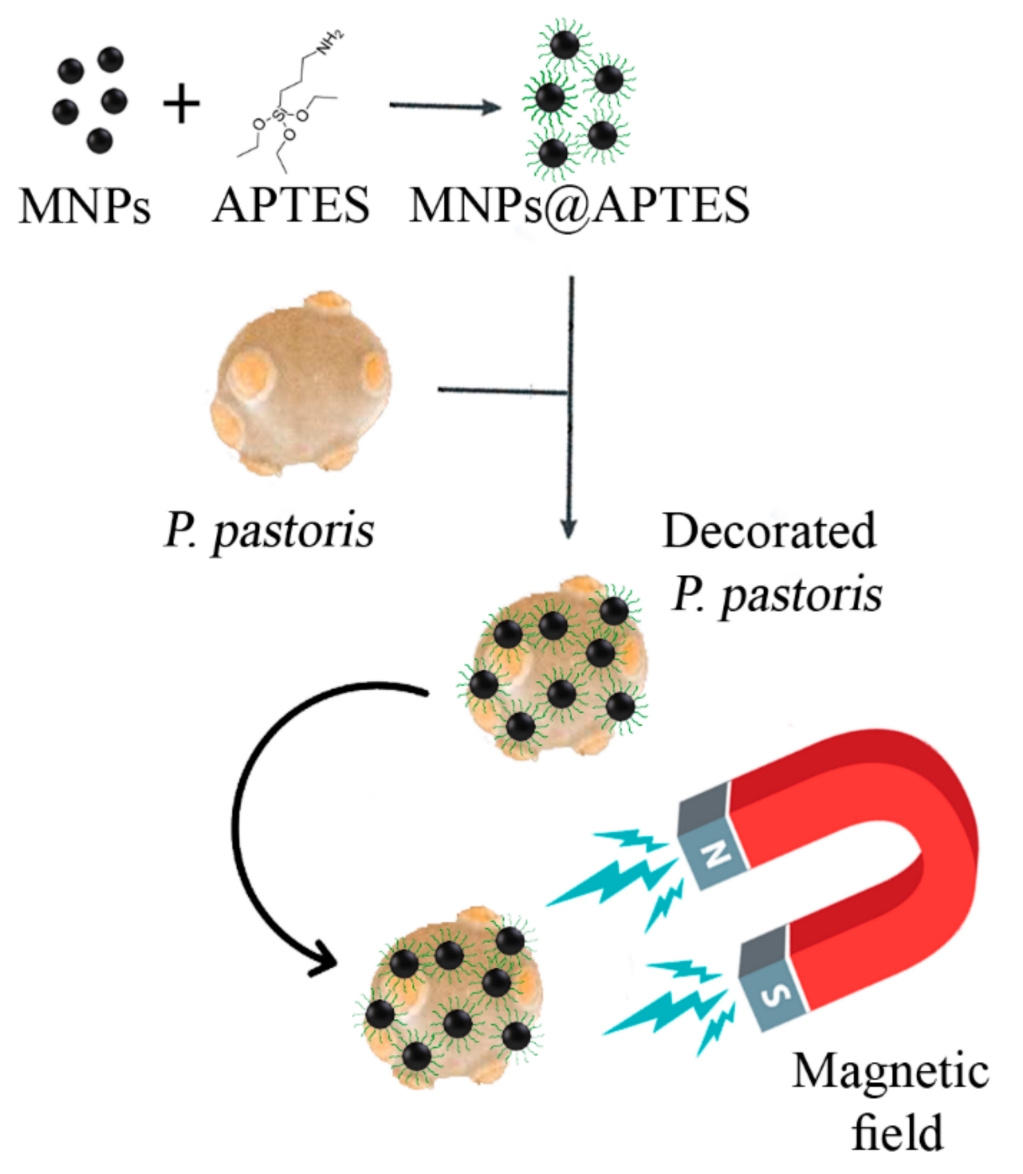
Decoration of *P. pastoris* cells with APTES-coated MNPs (MNPs@APTES) to make the cells magneto-responsible.

**Figure 7:**
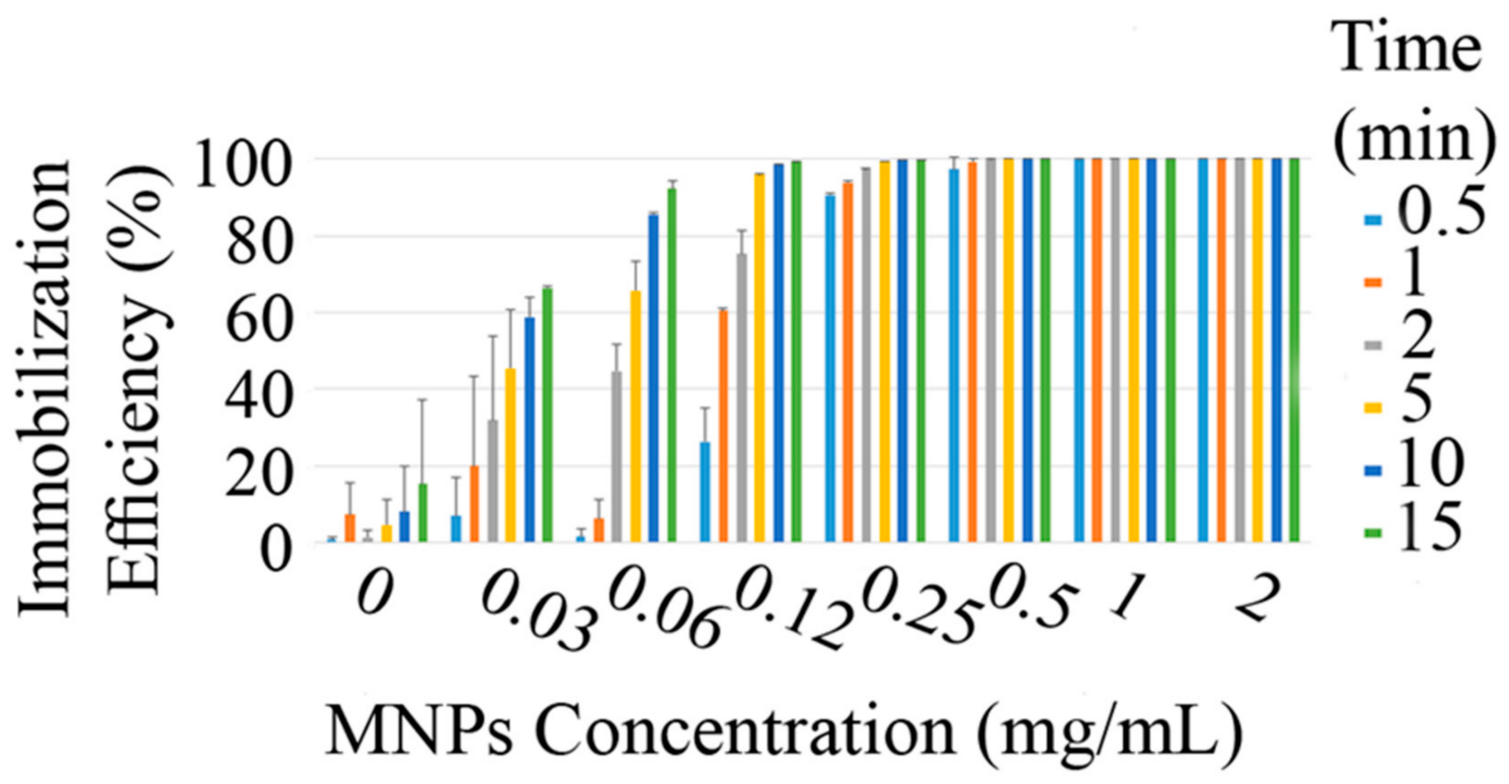
Efficiency of the magnetic immobilization (E_i_) in various concentrations of MNPs and different time periods of exposure to an external magnetic field with a Tesla rating of 1.4.

Previous investigations indicated that MNPs can interact with the eukaryotic and prokaryotic microbial cells’ surface via nonspecific forces. These particles can decorate microbial cells and make them magneto-responsible. So, decorated cells can be easily harvested and removed by employing an external magnetic field (Scheme 1). To evaluate the potential of prepared nanoparticles to immobilize and separate *P. pastoris* cells, various concentrations of MNPs were tested. Immobilized cells were exposed to a magnetic field in various time periods, from 30 s to 15 min. The calculated values of the immobilization efficiency (E_i_) are depicted in the Figure 7. The same experiments were also performed on the free cells (0 mg/mL MNPs) to evaluate the rate of cell sedimentation based on gravity. The results indicated that E_i_ is in close relation with the nanoparticle concentration, and from an increase in the MNP concentration, a significant increase in E_i_ occurred. At low concentrations (≤250 µg/mL), the time of the exposure to the magnetic field is another significant parameter, and by increasing the exposure period, more cells were attracted to the external magnet. Complete cell recovery (E_i_ = 100) was achieved in a very short time of exposure to the magnetic field by using ≥0.5 mg/mL MNPs.

### 3.3. Recombinant HSA Production

**Figure 8:**
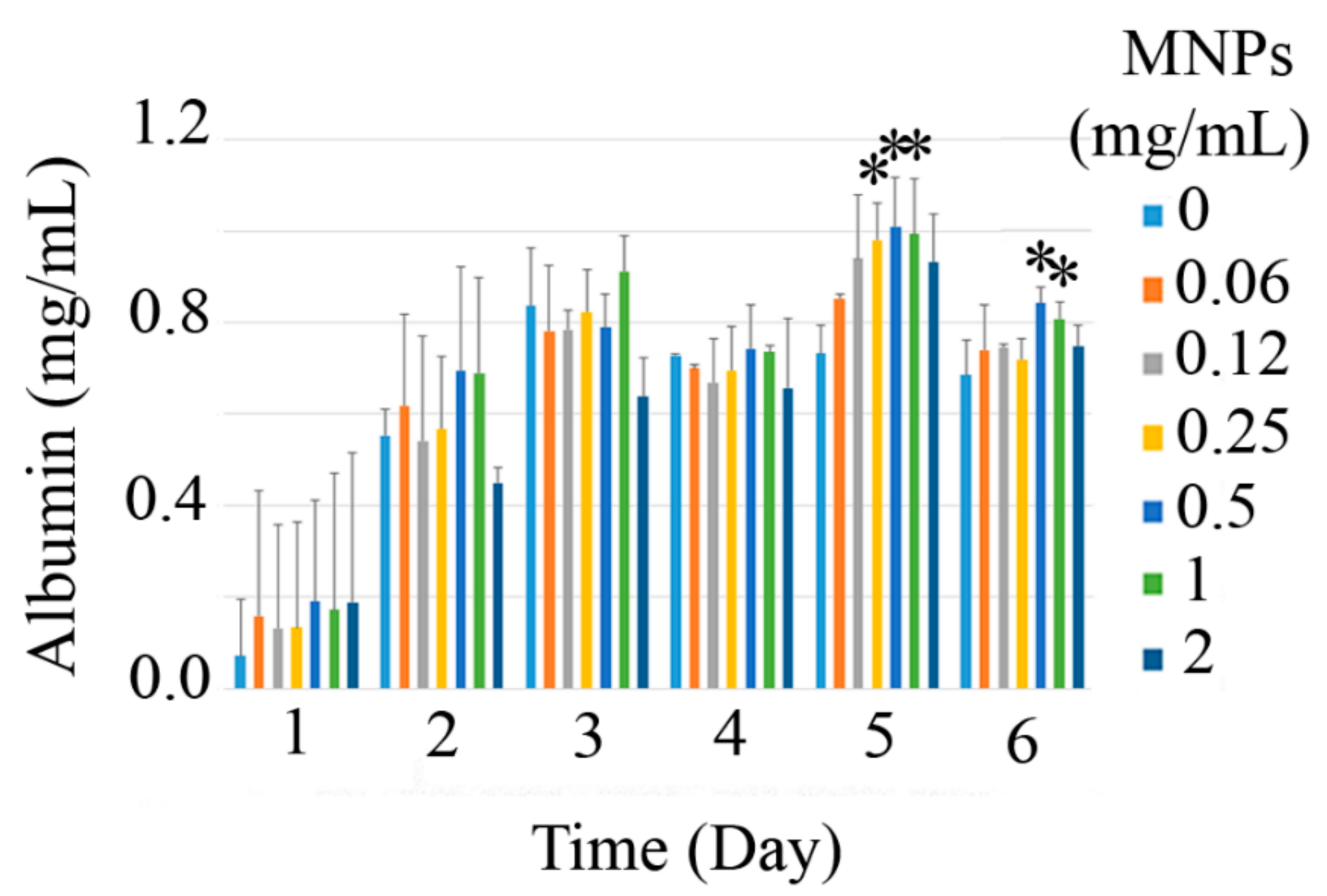
Recombinant HSA production in *P. pastoris* cells through different days of induction at various concentration of MNPs (0 to 2 mg/mL), stars indicate the significant (*p* < 0.05) difference with the control (0 mg/mL MNPs) in each day.

The employed *P. pastoris* cells for the production of recombinant HSA were strain GS115 Albumin (genotype His4, phenotype Mut^s^), which has an *HSA* gene under the high-inducible P*_AOX1_* promoter. The promoter is induced by methanol, and albumin production occurred over 5 days. In this experiment, the potential of the free and immobilized *P. pastoris* cells for HSA production was monitored over 6 days of induction with methanol, and the results are depicted in Figure 8. It can be seen that HSA production is completed after 5 days of induction in all cells. Compared to the free cells, magnetic immobilization with 250 μg/mL to 1 mg/mL nanoparticles increased the efficiency of *P. pastoris* cells for HSA production (*p* < 0.05). The largest increase in HSA production (38.1%) was achieved by the cells that were immobilized with 500 μg/mL of nanoparticles. This is the first time that we can achieve a higher production in the immobilized *P. pastoris*. In previous experiments, cells were immobilized in a polymeric matrix, and a significant reduction in cell performance and production was inevitable [12,13,15,16].

### 3.4. Nanoparticles’ Interaction with Cells

**Figure 9:**
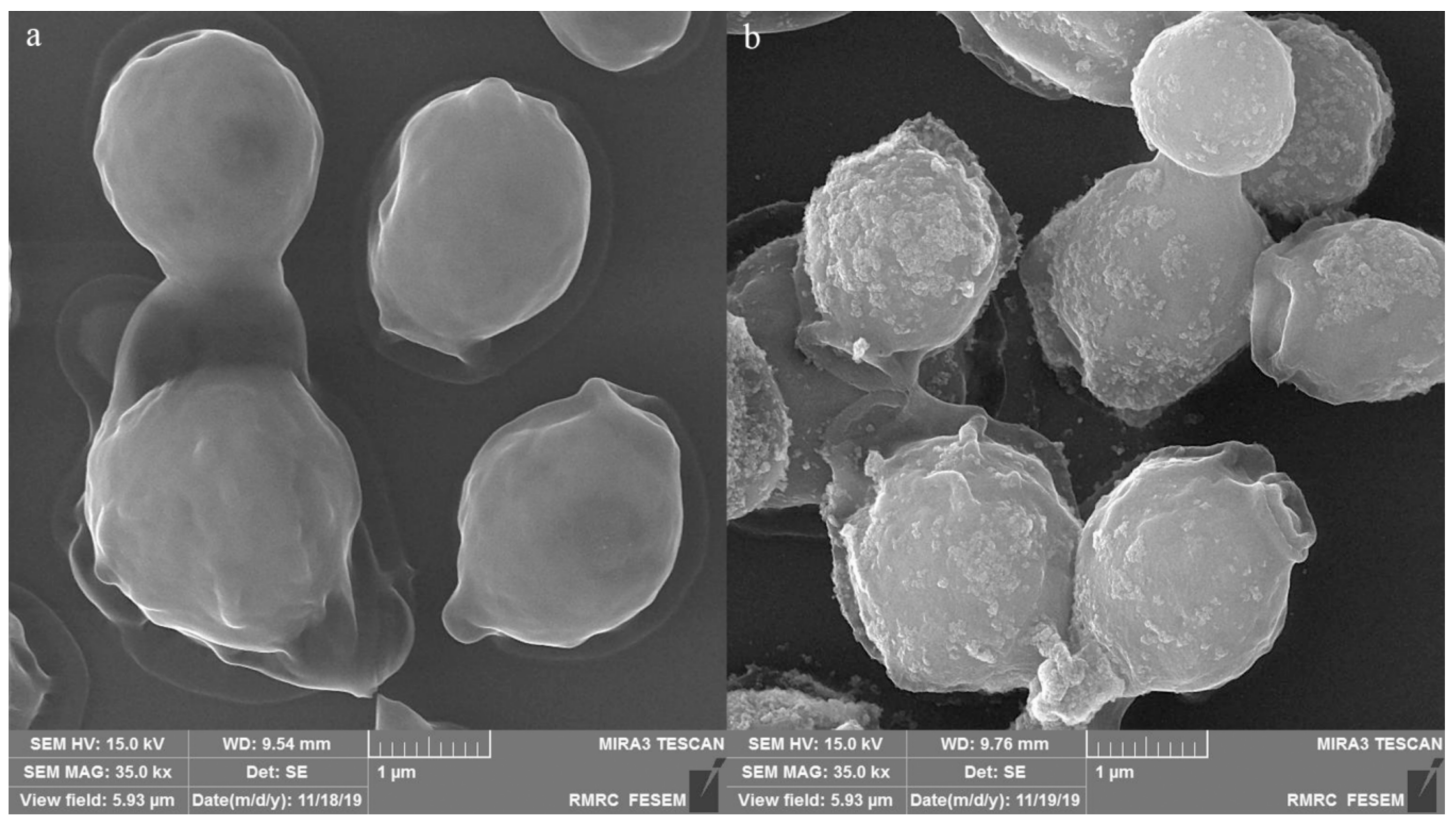
SEM micrographs of the free (**a**) and immobilized (**b**) *P. pastoris* cells that indicate the potency of the prepared MNPs for yeast cells’ decoration.

The interaction of MNPs with *P. pastoris* cells was visualized by SEM analysis, and the micrographs are provided as Figure 9. Free cells can be seen by the smooth surface (Figure 9a) while cells decorated with MNPs provide a rough surface appearance for the cells (Figure 9b). These results revealed the potency of the prepared nanoparticles to immobilize yeast cells efficiently.

## 4. Discussion

This is the first report that has employed magnetic immobilization for recombinant protein production in a eukaryotic host. The results were in agreement with previous reports for magnetic immobilization of bacterial cells [21,22,25]. MNPs can interact with prokaryotic and eukaryotic cells surface via nonspecific interactions. It has been shown that the decoration of microbial cells with these nanoparticles efficiently makes the cells sensitive to a magnetic field. This phenomenon is the basis for magnetic immobilization and magnetic separation.

The potential of the nanoparticles to separate cells is affected by various parameters, such as the power of the external magnetic field, magnetic saturation value of the employed particles, nanoparticles zeta potential, time of exposure to the magnetic field, and ratio of nanoparticles to the cells. So, in this experiment, MNPs were functionalized by amino groups in the APTES molecule. However, this coating did not provide a significant positive charge on the particles, as the zeta potential was measured to be 8.28 mV. It is obvious that a large positive zeta potential would increase the electrostatic interactions with the cells’ surface. However, there are some other reports that show that nanoparticles with negative surface charges can effectively decorate and separate microbial cells [32]. In contrast to previous reports, a much larger magnetic field was applied to achieve higher E_i_ in a short period of time. An external magnetic field with a gauss rating from 250 to 800 was employed in other reports [21,25,27]. While in this experiment, a neodymium magnetic with a Tesla rating of 1.4 was employed. Also, it should be clarified that the expression in the *P. pastoris* system is performed at extremely high cell densities (OD ~20) and this is the first time that magnetic immobilization was successfully used to harvest such a thick cell suspension.

Previous investigations showed that the decoration of bacterial cells with MNPs disturbs the cytoplasmic membrane integrity due to the penetration of nanoparticles in the cell membrane. The disturbed membrane cannot efficiently manage mass transfer and hence there is an increased permeability across cell barriers. To some extent, an increase in the membrane permeability can increase the cells’ performance in biotechnological processes. This effect was reported by F. Ansari and her colleague in 2009 when working on a dibenzothiophene (DBT) degrading strain of *Rhodococcus erythropolis*. They found that decoration of the bacterial cells with glycine-coated MNPs made their membrane more permeable and enhanced DBT degradation [24]. Similar results were reported for a recombinant strain of *Escherichia coli* that produces extracellular asparaginase (II). The authors showed that decorated cells can secrete more enzyme than free cells [25].

In the case of yeast cells, they have a unique cell wall, which differs with the cell wall in other cells. It is a rigid structure about 100 to 200 nm thick and constituting about 25% of the total dry mass of the cell. Yeasts’ cell wall composition is vastly changed in different growth conditions. However, in general, it mainly consists of proteins and polysaccharides and is composed of four classes of macromolecules, including chitin, mannoproteins (a highly glycosylated glycoproteins), and two types of beta glucans. These biomolecules provide carboxyl, amino, and hydroxyl moieties that can anchor metals on the cell surface [39]. On the other hand, nonspecific interactions, such as electrostatic forces, hydrogen bonds, hydrophobic interactions, and weak Van der Waals bonds, can be considered for the attachment of nanoparticles on the living cells’ surface. These forces are sufficient to keep the small nanoparticles on the cell surface [25,27]. Also, it is approved that metal nanoparticles are not just attached to the surface of the cells. Particularly, in the case of *P. pastoris*, it has been shown that these cells can stably immobilize metal nanoparticles in their envelope [39]. All the particles that interact with the cell, either on the cell surface or inside the cell, would provide force in the same direction toward the magnetic field and make the cell magneto-responsible. Like bacterial cells, it is possible that the penetration of nanoparticles in the yeast cell envelope is the reason for an increased HAS production in immobilized *P. pastoris* cells. It may be considered that the penetration of MNPs into the *P. pastoris* cells or the attachment of nanoparticles to the cell receptors may have some effects on the gene expression regulations and HSA production. However, in fact, it is not the case. In our preliminary investigations, we found that MNPs had no inducing effect on recombinant *P. pastoris* cells and no HSA was produced in the absence of methanol. According to our best knowledge, this is the first report that shows that magnetic immobilization can increase the biotechnological performance of *P. pastoris*.

## 5. Conclusions

Magnetic immobilization was introduced as a promising approach to overcome defects in common polymer-based cell immobilization techniques. This novel approach has been employed for the production of valuable products in a few bacterial cells and interesting results were reported. The *P. pastoris* system is selected as a standard and developing tool in recombinant protein production. *P. pastoris* GS115 Albumin was employed to produce extracellular recombinant HSA. The results revealed that amine-functionalized MNPs can efficiently decorate yeast cells and make the cells magneto-responsible. The sensitivity of decorated cells to an external magnetic field was extremely high. In some concentrations (≥1 mg/mL), just 30 s of exposure to the magnet was sufficient to remove all the cells. It is worth mentioning that in the *P. pastoris* system, expression occurred in a very high cell density (OD ~20). It is interesting that the applied technique can successfully harvest all the cells from such a thick suspension. Another interesting feature of this experiment was the enhanced HSA production in the immobilized cells in contrast to free cells. These data are promising and introduced a new avenue for future experiments in cell immobilization and recombinant protein production in eukaryotic systems.
